# Cardiac Autonomic Modulation and the Kinetics of Heart Rate Responses in the On- and Off-Transient during Exercise in Women with Metabolic Syndrome

**DOI:** 10.3389/fphys.2017.00542

**Published:** 2017-07-26

**Authors:** Lucas R. B. E. Silva, Antonio R. Zamunér, Paulo Gentil, Fagner M. Alves, Acácia G. F. Leal, Viviane Soares, Maria S. Silva, Marcus F. Vieira, Karina Simões, Gustavo R. Pedrino, Ana C. S. Rebelo

**Affiliations:** ^1^School of Medicine, Federal University of Goiás Goiânia, Brazil; ^2^Department of Physical Therapy, Sacred Heart University Bauru, Brazil; ^3^School of Physical Education and Dance, Federal University of Goiás Goiânia, Brazil; ^4^Department of Morphology, Biological Sciences Institute, Federal University of Goiás Goiânia, Brazil; ^5^Evangelical Educational Association, University Centre of Anápolis Anápolis, Brazil; ^6^Center of Neuroscience and Cardiovascular Research, Federal University of Goiás Goiânia, Brazil

**Keywords:** autonomic dysfunction, fasting glucose, acute and late complications, high intensity interval exercise, oxygen consumption

## Abstract

**Objective:** To test whether women with metabolic syndrome (MS) have impairments in the on- and off-transients during an incremental test and to study whether any of the MS components are independently associated with the observed responses.

**Research Design and Methods:** Thirty-six women aged 35–55 years were divided into a group with MS (MSG, *n* = 19) and a control group (CG, *n* = 17). R-R intervals (RRi) and heart rate variability (HRV) were calculated on a beat-to-beat basis and the heart rate (HR) at the on- and off-transient were analyzed during an incremental cardiopulmonary exercise test (CPET).

**Results:** MSG showed lower aerobic capacity and lower parasympathetic cardiac modulation at rest compared with CG. HR values in on-transient phase were significantly lower in MSG compared with CG. The exponential amplitudes “amp” and the parameters “τ” [speed of heart rate recovery (HRR)] were lower in MSG. MSG exhibited higher HR values in comparison to CG during the off-transient indicating a slower HRR. In MSG, there was an inverse and significant correlation between fasting plasma vs. ΔF and glucose vs. exponential “τ” of HRR dynamics.

**Conclusion:** MS is associated with poor heart rate kinetics. The altered HR kinetics seems to be related to alterations in cardiac parasympathetic modulation, and glucose metabolism seems to be the major determinant.

## Introduction

Metabolic syndrome (MS) is a complex trait characterized by a cluster of interconnected factors, including central obesity, glucose intolerance, elevated blood pressure and dyslipidemia (Alberti et al., 2009). Among the behavioral approaches indicated to treat and prevent MS, physical activity (Golbidi et al., 2012; Roberts et al., 2013; De Sousa and Norman, 2016), especially high intensity exercise (Aguilera Eguia et al., 2015; Baldi et al., 2016), has gained increased attention. However, physical exertion requires cardiovascular adjustments that might be impaired in some diseases, increasing the potential risk for cardiovascular events. Therefore, understanding cardiovascular responses to exercise is of great value for screening to identify cardiovascular risks and increase safety during exercise prescription.

Cardiovascular adjustments occurring both during rest-exercise transition (on-transient) and exercise-recovery transition (off-transient) have been shown to provide relevant clinical information (Javorka et al., 2003). The on- and off-transients reflect changes in cardiac autonomic modulation to cope with the energy demands required by exercise. During the on-transient, there is a combination of parasympathetic withdrawal and sympathetic activation (Mitchell, 1985; Sietsema et al., 1989), while the off-transient is mainly thought to be dependent on parasympathetic nervous system reactivation (Arai et al., 1989; Imai et al., 1994). Abnormalities in cardiovascular and metabolic off-transient have been reported in coronary heart disease (Spies et al., 2005), obesity/overweight (Franco et al., 2015; Tomlinson et al., 2015), diabetes (Baldi et al., 2016), aging (Simões et al., 2013), and have been consistently related to cardiovascular risk and increased mortality (Cole et al., 2000; Nishime et al., 2000; Watanabe et al., 2001; Javorka et al., 2003). The higher resting heart rate (HR) in people with insulin resistance (Baldi et al., 2016) is supposed to be a compensatory mechanism adopted to maintain cardiac output in face of a lower stroke volume (Gusso et al., 2008; Pinto et al., 2014). Although MS shares many clinical symptoms with diabetes, it is not known whether such alterations are also found in MS. Moreover, it remains unknown if such alterations are due to a decrease in sympathetic, an increase in parasympathetic modulation or both.

The increase in HR in response to exercise is attenuated in diabetes (Gusso et al., 2008; Pinto et al., 2014; Baldi et al., 2016), which contrasts with the elevated sympathetic and attenuated parasympathetic activity and suggests that the autonomic modulation during exercise might be different from rest. Whilst the off-transient is commonly studied in different populations, the on-transient might also be an important parameter as this reflects the cardiac adjustments adopted to supply the energy demands of the working muscles (Rowell and O'Leary, 1990). In this regard, a slowed parasympathetic withdrawal might result in a slowed on-transient and can ultimately impair exercise performance due to an inadequate blood supply to the working muscles. However, an increased sympathetic modulation would induce a high HR response at the start of the exercise, leading to a disproportionately high cardiovascular stress in response to physical exertion. Therefore, knowing the alterations in on-transient might be important to understand the HR response to exercise in MS patients, enabling the adequate design and control of exercise programs in this population.

Another issue that remains to be clarified is whether MS itself is a risk factor for cardiovascular disease (that is, the sum of individual components that constitute the syndrome) or whether one of the risk factors, individually, is capable of compromising neurocardiac integrity (Kahn et al., 2005). Previous studies have suggested that the MS itself, and not merely a single component, is related to autonomic dysfunction (Spies et al., 2005; Deniz et al., 2007; Alihanoglu et al., 2015). However, impaired vagal reactivation and sympathetic overactivity are known to be associated with hyperinsulinemia and insulin resistance (Baldi et al., 2016), suggesting that glucose metabolism might play a central role in MS (Gingras et al., 2017). In addition, increased waist circumference has been consistently associated with cardiovascular risk (Ross et al., 2008; WHO, 2011), probably due to the release of pro-inflammatory cytokines by visceral adipose tissue (Gaggini et al., 2015). Previous data suggest also that increase in fat mass is strongly associated with increase in sympathetic and decrease in parasympathetic modulation, both at rest and during exercise (Aimbire et al., 2006). Moreover, a factorial analysis indicated that adiposity accounts for the unique feature of MS (Anderson et al., 2001). To the best of our knowledge, the association between these factors and the cardiovascular responses to exercise has not been established in MS.

It is important to better understand the cardiovascular adjustments during the rest-exercise and exercise-recovery transitions in people with MS and also to study the factors associated with such changes in order to adequately treat and manage MS. Therefore, the present study aimed to test the hypothesis that women with MS have impairment in the HR response during on- and off-transients during an incremental test and to assess whether any of the MS components are independently associated with the observed responses.

## Research design and methods

### Participants

The present study was a controlled cross-sectional study. Thirty-six women aged 35–55 years were recruited and allocated into two groups. One group included 19 women with MS (MSG). Seventeen healthy women, matched for age to MSG, were allocated to the control group (CG).

Exclusion criteria included skeletal muscle and/or joint pain, and cardiopulmonary and neurological diseases, or difficulty in performing the exercise protocol used in the study. The use of antihypertensive drugs, such as angiotensin-converting enzyme inhibitors and diuretics (87.5% in MS group and 4.7% in CG group), antidepressants (33.3% in MS group and 19%, anti-hypoglycemic agents (20.8% in MS group), antihypertriglyceridemic (8.3% in GMS), antihypothyroidism (12.5% in MS group and 4.7% in CG group) was reported by the volunteers. In the MS group, 36.8% of the volunteers reported being in the climacteric period, while in the CG group 10% were in this period.

This study was carried out in accordance with the recommendations of the ethics committee of institution with written informed consent from all subjects. All subjects gave written informed consent in accordance with the Declaration of Helsinki. The number of the approved protocol was 784446/14.

### Assessment protocols

The participants were tested in the morning period (7–10 a.m.) to minimize circadian influences. Room temperature was maintained at 22°C with relative air humidity between 50 and 60%. Participants were informed about the experimental protocol and instructed to abstain from use of stimulants (coffee or tea) and alcoholic beverages during the 24 h preceding the test, as well as, to have a light meal at least 2 h before testing.

All participants attended the laboratory on 2 occasions. The first visit occurred 1 week before the test and the participants were screened for eligibility and were acquainted with the experimental procedures. On the second visit, the participants were interviewed and examined before testing to confirm they were in good health, to establish whether they had slept well the previous night and whether they had complied with the instructions. Participants' body weight (BW) was measured using a digital scale, and their height was measured with a portable stadiometer. Body mass index (BMI) was calculated as the ratio of the BW (kg) to the square of the height (m^2^). Volunteers were evaluated between 7 and 10 days after the start of menses. Volunteers who reported use of oral contraceptives (with 21 days of active pills followed by 7 days of placebo) for at least 18 months were included, and the evaluation occurred during the placebo phase. Waist circumference (WC) was measured with an anthropometric tape at the midpoint between the iliac crest and the last rib at the end of expiration while the participant was at rest.

HR and blood pressure were measured after 5 min of rest, in the supine and sitting positions, by the Korotkoff auscultatory method and every 2 min during the test period with a mercury column sphygmomanometer and a stethoscope (Littman, St. Paul, MN, USA). The diagnosis of MS was established based on increased waist circumference (≥80 cm) and the presence of at least two of the following criteria: elevated triglycerides (≥150 mg/dL) or treatment for dyslipidemia, reduced HDL (<50 mg/dL) or treatment for this abnormality, elevated blood pressure (systolic arterial pressure ≥130 mmHg and diastolic arterial pressure ≥85 mmHg) or treatment for hypertension and elevated fasting glucose (≥100 mg/dL) or treatment for hyperglycemia (Alberti et al., 2009). Blood collection was performed by trained professionals in the morning (7–9 h). After collection, the samples were immediately conditioned in a container with adequate temperature and sent for analysis.

The participants were instructed not to talk during the assessment to avoid interfering with the electrocardiogram signal and to communicate any change in their overall state before, during or after protocol application. HR was continuously recorded, on a beat-to-beat basis, during the entire protocol, i.e., before testing, during cardiopulmonary exercise testing (CPET) and 6 min after the end of exercise.

### Cardiopulmonary exercise testing

Cardiorespiratory fitness was assessed by an incremental ergospirometric test using a Centurion 200 electronic treadmill coupled to a portable computer. The modified Bruce's protocol was used for this study. Participants remained at rest in the orthostatic position for 3 min pre-exercise, followed by a 2-min warm up at 5 km/h. Speed was increased by 1 km/h every minute until exhaustion. After exhaustion active recovery was performed for 2 min at 2 km/h and the participant then sat for 4 min. The criteria used for test interruption were: (1) incapacity of the participant to perform the exercise; (2) accentuated increase in systolic arterial pressure (reaching values greater than 200 mmHg); (3) reaching maximum age-predicted HR (Fox and Haskell, 1968) and (4) respiratory exchange ratio >1.15.

HR was continuously monitored using a HR monitor (Polar V800, Finland). Blood pressure was measured every 2 min using a mercury sphygmomanometer. Ratings of perceived exertion (RPE) were assessed using the Borg Scale at the end of each stage of the exercise protocol. This scale ranges from 6 (rest) to 20 (maximum intensity). The participant reported RPE separately for legs and dyspnea. The expired air was continuously measured breath-by-breath using a portable gas analyzer (Cortex, Metalyzer II, Rome, Italy). The criteria for cardiorespiratory fitness classification based on peak oxygen uptake followed the American Heart Association (AHA) recommendations (AHA, 1972).

### RR recording and HR variability (HRV) analysis

R-R intervals (RRi) were recorded at rest over a 12-min period using a cardiofrequencimeter (Polar® V800, Oi, Finland), while the volunteers were seated and breathing spontaneously. HRV was analyzed by linear (time and frequency domains) and non-linear (Shannon Entropy) methods. The region of greatest stability of the RRi time series with 256 consecutive beats was selected for the analyses.

Time domain parameters studied were the standard deviation of all RRi (SDNN) and the square root of the mean squared differences between adjacent RRi (rMSSD). SDNN reflects overall HRV, whereas rMSSD is considered to be an index of cardiac parasympathetic modulation. For frequency domain, spectral analysis was performed using Fast Fourier Transformation applied to a single window after a linear trend subtraction at the previously chosen RRi. The spectral components were obtained at low frequency (LF: 0.04–0.15 Hz) and high frequency (HF: 0.15–0.4 Hz) in absolute units (ms^2^), and the normalized units were computed by dividing the absolute power of a given LF or HF component (ms^2^) by the total power minus very low frequency (0.003–0.04 Hz) power and then multiplying this ratio by 100. Since the LF band is modulated by both the sympathetic and the parasympathetic nervous systems and the HF band is correlated with vagal cardiac control, the LF/HF ratio was calculated to determine the sympathovagal balance.

### On-transient

The HR data obtained during the CPET were filtered and entered into MatLab for analysis. The model used for fitting the kinetic response in the rest-exercise transition involved 60s of rest condition plus the first 180s of exercise. For analysis, the first 20s of data were discarded. ΔT is the time constant which represents the time of vagal withdrawal with the increase of load at the beginning of the physical exercise. ΔF reflects the amplitude of the HR response at the beginning of physical exercise, which is computed subtracting from the HR of the first peak (vagal withdrawal) the mean value of HR calculated in the first 60s of rest condition (Simões et al., 2013).

### Off-transient

The HR data at the off-transient were collected at the end of CPET and then filtered and analyzed with an *ad hoc* routine developed using OriginPro 8.0 software (OriginLab, Northampton, MA, USA). This algorithm applies an exponential model to the data corresponding to the full recovery period (2 min cool-down and 4 min of rest; Imai et al., 1994). A non-linear algorithm that minimizes the sum of squared errors as a convergence criterion was used to determine the best parameters for the resulting exponential curve (Motulsky and Ransnas, 1987). The function was only included in the final analysis if *r* > 0.95. The off-kinetics were modulated using the following time exponential function (Rossiter et al., 2002):

(1) $$\begin{array}{r} \text{HR}\left(\text{t}\right)={\text{HR}}_{\text{peak}}-{\text{a}}^{*}\left({1-\text{e}}^{-\left(\text{t}-\text{TD}\right)/\tau }\right) \end{array}$$

where “t” is time, “HR_peak_” is the peak HR at the end of CPET, “A” is the amplitude of HR reduction after the end of exercise, “τ” is the exponential time constant, and “TD” is a time delay. The inclusion of “TD” was due to the possibility of HR_off_ not decreasing immediately after load interruption.

### Statistical analysis

Descriptive analyses were performed to characterize the sociodemographic data. The normality of data distribution was assessed by means of the Kolmogorov-Smirnov test. Differences between the two independent groups were assessed using the *t*-test for independent samples. Multivariate analysis of variance (MANOVA) was used to assess differences between the two groups (with and without MS) with respect to the various times at which HR was evaluated. Differences between groups were also assessed by an analysis of covariance (ANCOVA) considering age and being/not being on climacteric period as covariates. Since the results remained unchanged, they were not presented. A stepwise linear regression was performed to identify whether MS components could predict the results of HRV at rest, and in the kinetics of on- and off-transients. Dependent variables included HRV indices (SDNN, rMSSD, HF, LF, and HF/LF) and HR kinetics parameters (ΔT, ΔF, amp, and τ). Independent variables were MS components (waist circumference, fasting blood glucose, HDL, SAP, DAP, and triglycerides). The chi-square test was used to investigate the association between categorical variables, the presence (or not) of MS and VO_2_peak. Significance level was set at 5%. The statistical analyses were performed using the Statistical Package for the Social Sciences, version 15 (SPSS).

## Results

Baseline clinical characteristics of the MSG and CG groups are presented in Table 1. There was no significant difference between the two groups regarding age and height (*p* > 0.05). As expected, patients with MS had higher BW, BMI, and WC. Also as expected, regarding hemodynamic variables, MSG presented higher systolic and diastolic blood pressure compared with CG (*p* < 0.05). No significant difference was found for resting HR (*p* > 0.05).

**Table 1:** Anthropometric characteristics, cardiovascular risk factors, cardiorespiratory fitness, HR variability and kinetics of the volunteers evaluated.

	**CG (*n* = 17)**	**MSG (*n* = 19)**	***p***
**ANTHROPOMETRIC CHARACTERISTICS**			
Age, years	38.57 ± 8.22	43.62 ± 8.51	0.063^a^
Stature	1.60 ± 0.06	1.56 ± 0.05	0.069^a^
Weight, kg	77.86 ± 11.54	89.75 ± 19.56	0.010^b^
BMI, kg/m^2^	30.27 ± 3.99	36.56 ± 7.62	< 0.001^b^
**CARDIOVASCULAR RISK FACTORS**			
Waist circumference, cm	90.88 ± 12.81	107.47 ± 17.33	< 0.001^a^^*^
PAS, supine rest, mmHg	114.05 ± 10.22	125.86 ± 16.03	< 0.005^a^^*^
PAD, supine rest, mmHg	75.15 ± 7.12	84.56 ± 11.22	< 0.001^b^^*^
HDL-c, mg/dL	43.64 ± 8.87	41.63 ± 5.82	0.188^b^
Triglycerides, mg/dL	90.52 ± 26.40	163.73 ± 66.31	< 0.001^b^^*^
Fasting glucose, mg/dL	89.76 ± 6.24	126.84 ± 75.55	< 0.001^b^^*^
**CARDIORRESPIRATORY FITNESS**			
HR pre-exercise rest, bpm	83.47 ± 6.77	87.31 ± 17.42	0.286^b^
VO_2peak_, mL/min/kg	21.94 ± 5.09	18.68 ± 5.50	0.037^a^^*^
VCO_2_, mL/min/kg	17.51 ± 5.71	14.93 ± 5.37	0.190^a^
Test Time, min	6.54 ± 2.10	4.60 ± 2.15	0.009^a^^*^
Distance traveled, m	735.45 ± 221.69	560.86 ± 206.54	0.007^a^^*^
VO_2_/WorkLoad	0.26 ± 0.05	0.22 ± 0.08	0.075^a^
VO_2_/HR	0.35 ± 0.05	0.39 ± 0.16	0.346^b^
RER	1.15 ± 0.02	1.14 ± 0.03	0.873^a^
Borg scale-breathing	14.66 ± 3.09	15.09 ± 2.14	0.598^a^
Borg scale-peripheral	12.23 ± 2.57	16.45 ± 2.09	0.036^a^^*^
**AUTONOMIC MODULATION**			
SDNN (ms)	35.24 ± 13.49	26.02 ± 11.02	0.030^a^^*^
rMSSD (ms)	25.12 ± 12.62	17.03 ± 8.87	0.032^b^^*^
HF (un)	0.18 ± 0.25	0.08 ± 0.11	0.004^a^^*^
LF (un)	0.28 ± 0.05	0.28 ± 0.06	0.482
HF/LF	1.42 ± 1.26	3.18 ± 3.34	0.044^b^^*^
**HR KINETICS**			
On-transient			
ΔT, sec	33.00 ± 19.47	103.11 ± 18.75	<0.0001^b^^*^
ΔF, degree	17.32 ± 6.87	19.51 ± 10.00	0.209^b^
**Off-transient**			
Amp, degree	60.02 ± 14.62	44.01 ± 16.07	0.002^a^^*^
HRpeak, bpm	169.73 ± 15.87	167.95.±23.33	0.414^a^
τ, sec	96.24 ± 16.59	178.83 ± 91.86	0.036^b^^*^

Data are presented as mean ± standard deviation. MSG, metabolic syndrome group; CG, control group; HR, heart rate; BMI, body mass index; SBP, systolic blood pressure; PAD, diastolic blood pressure; HDL-c, high-density lipoprotein cholesterol; VO2peak, peak oxygen consumption; VCO_2_, carbon dioxide production; RER, respiratory exchange rate; SDNN, standard deviation of all normal RR intervals recorded in a time interval, expressed in milliseconds (ms); RMSSD, square root of the mean squared differences between the adjacent normal RR intervals, in a time interval, expressed in ms; HF, high frequency (un); LF, low frequency (un); HF/LF, ratio; ΔT, time constant; ΔF, frequency constant; Amp, amplitude. τ, time constant.;

a T-test for independent samples;

b *Mann-Whitney test*.

* *Statistically significant (p < 0.05)*.

### Laboratory tests

MSG presented higher fasting plasma glucose, serum triglyceride and LDL compared with the control subjects. No significant difference was found for HDL.

### Cardiopulmonary exercise testing results

CG had higher VO_2_peak, test time and distance. There was no significant difference between groups with regard to dyspnea RPE. However, patients with MS reported higher leg RPE compared with the CG subjects at the test peak.

### HRV analysis

CG group showed higher cardiac parasympathetic modulation at rest compared with CG, as indicated by higher values of SDNN, rMSSD, HF and lower HF/LF ratio. There was no significant difference between groups regarding LF and Shannon Entropy (Table 1).

### Kinetics of HR responses

**Figure 1:**
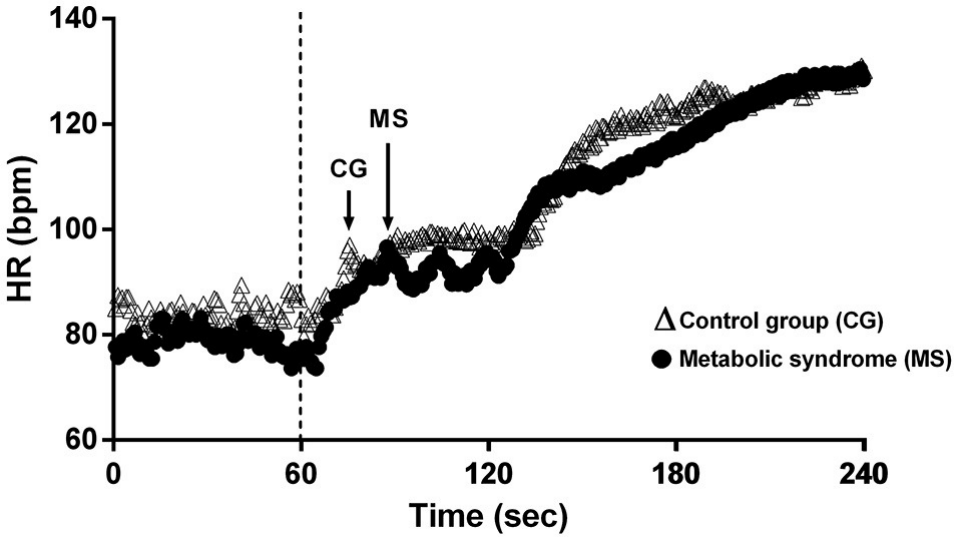
Illustration of heart rate on-transient in a participant from the control group (CG) and in a participant from the metabolic syndrome group (MSG). Dashed line represents the exercise onset. Arrows represent the heart peak after vagal withdrawal. See that the heart peak in the MS subject is delayed compared to the control subject.

The kinetics of HR responses are summarized in Table 1. HR values in on-transient phase were significantly lower in the MS group compared with the CG subjects (*p* < 0.0001) (Figure 1), which represents slowed time of vagal withdrawal with the increase of load at the beginning of the physical exercise (ΔT). There was no significant difference between the two groups in respect of ΔF, which reflects the angulation of the HR response at the beginning of physical exercise (Table 1).

**Figure 2:**
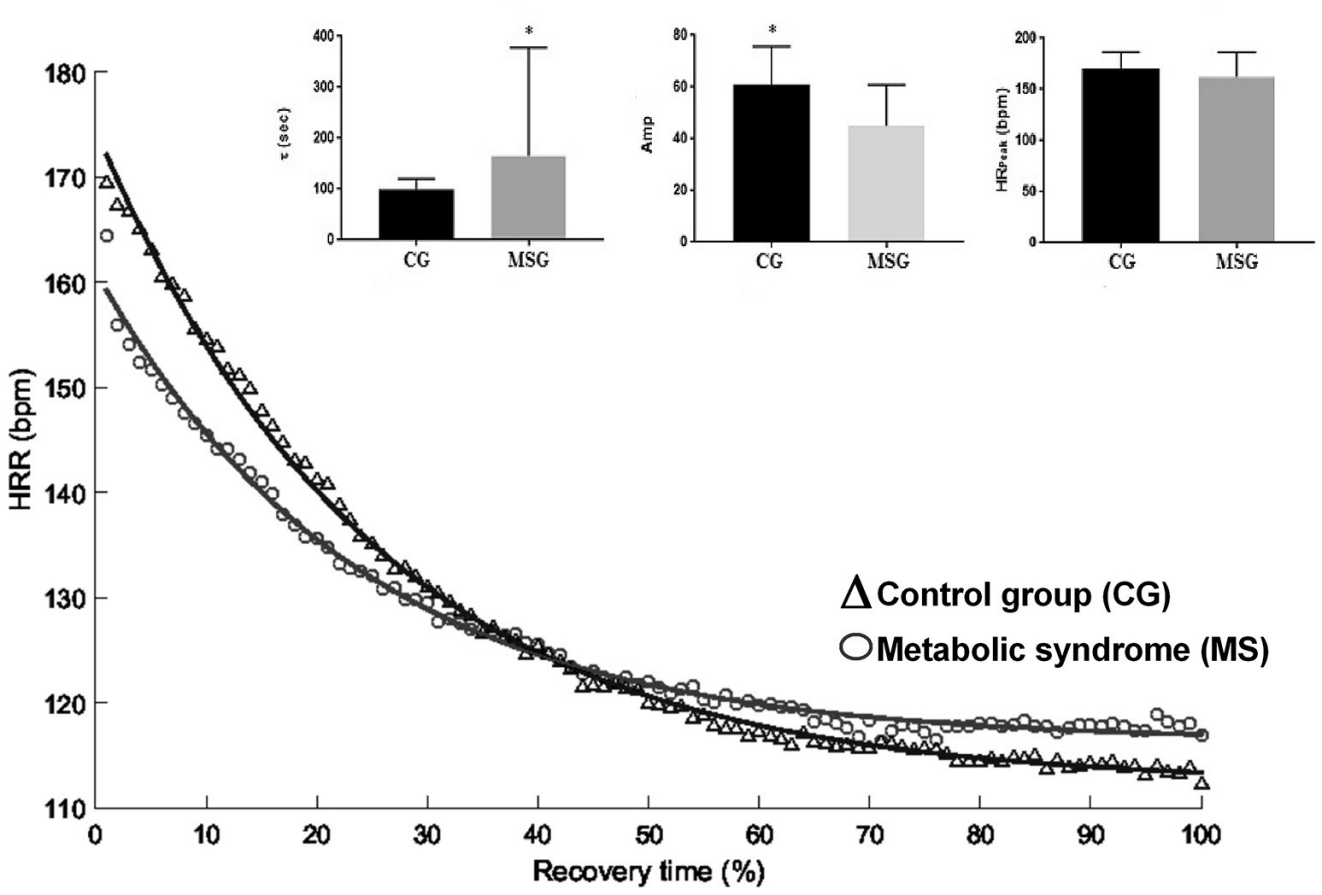
Comparison of peak and heart rate kinetic responses in the off-transient between groups with and without metabolic syndrome. CG, Control Group; MSG, Metabolic Syndrome Group; HRR, heart rate recovery; Amp, amplitude; HRpeak, heart rate peak; τ, time constant. *Significant different from CG (*p* < 0.05).

Mean HR recovery (HRR) exponential data for MSG and CG are shown in Table 1. HRR were significantly slower in MSG compared with the control group. The exponential amplitudes “amp” and the parameters “τ” (speed of HRR dynamics) were lower in CG (*p* < 0.005 and *p* = 0.03, respectively) as illustrated in Figure 2. “HR_peak_” did not differ between groups. The results of Δ analysis at the various intervals (15–120) showed that CG exhibited lower HR values in comparison with MSG for Δ_45_–Δ_120_, indicating a faster HRR dynamics during these intervals.

Table 2 shows the multiple regression analysis results for rest-exercise transition (on-transient) and exercise-recovery transition (off-transient). In MSG and CG there was a significant negative relationship between fasting plasma glucose and ΔF (β = −0.07; *r*^2^ = 0.14; *p* = 0.016). In the MS group, there was a significant negative relationship between fasting plasma glucose and the exponential “τ” of HRR dynamics (β = 2.30; *r*^2^ = 0.84; *p* ≤ 0.001). A significant negative correlation between waist circumference and cardiorespiratory fitness (β = −0.26; *r*^2^ = 0.53; *p* = 0.003) was also observed in MSG. There was no significant correlation between any components of MS and VO_2peak_. In CG there was a significant negative relationship between BMI values and VO_2peak_ (β = −0.74; *r*^2^ = 0.54; *p* = 0.0003).

**Table 2:** Age-adjusted linear regression analysis of on-and off-transients and cardiometabolic risk factors in GSM and GNSM.

	**CG (*n* = 17)**						**MSG (*n* = 19)**					
	**Δτ**			**ΔF**			**Δτ**			**ΔF**		
	**β**	***r*^2^**	***p***	**β**	***r*^2^**	***p***	**β**	***r*^2^**	***p***	**β**	***r*^2^**	***p***
WC	−0.053	0.012	0.783	−0.005	0.078	0.982	−0.026	0.000	0.940	−0.101	0.026	0.395
Glucose	−0.084	0.010	0.886	0.300	0.001	−0.299	−0.009	0.002	0.910	−0.077	0.144	0.016^*^
HDL	0.270	0.008	0.497	−0.299	0.175	0.162	1.209	0.077	0.271	−0.060	0.001	0.869
SBP	−1.633	0.247	0.300	−0.110	0.016	0.627	0.332	0.006	0.542	0.091	0.212	0.600
DBP	1.772	0.270	0.120	−0.343	0.068	0.277	−0.500	0.028	0.508	0.284	0.003	0.243
TGL	−0.252	0.162	0.107	0.072	0.026	0.351	−0.063	0.018	0.535	0.068	0.204	0.051
	**Amp**			**τ**			**Amp**			**τ**		
WC	0.322	0.009	0.266	−0.011	0.001	0.973	0.048	0.017	0.865	−1.831	0.016	0.209
Glucose	0.662	0.120	0.448	0.239	0.019	0.826	−0.051	0.124	0.489	2.302	0.839	< 0.001^*^
HDL	0.423	0.238	0.421	0.293	0.077	0.636	1.650	0.006	0.081	0.077	0.004	0.985
SBP	−0.314	0.013	0.670	0.150	0.011	0.872	0.082	0.003	0.827	−0.300	0.008	0.888
DBP	0.288	0.053	0.790	0.088	0.001	0.948	−0.164	0.002	0.783	3.476	0.014	0.252
TGL	−0.032	0.008	0.879	0.009	0.003	0.972	0.034	0.025	0.713	0.238	0.007	0.557

*CG, control group; MSG, metabolic syndrome group; N, number of participants; ΔT, time constant; ΔF, frequency constant. Amp, amplitude; τ, time constant; SBP, systolic Blood pressure; DPB, diastolic blood pressure; HDL-c, high-density lipoprotein cholesterol. TGL, triglycerides*.

* *statistically significant (p < 0.05)*.

## Discussion

The main findings of the present study were that women with MS have poor HR kinetics when compared with women without MS. Moreover, cardiac parasympathetic modulation impairment is suggested to be the main variable underlying such alterations and fasting glucose levels were the only independent variable explaining the impaired cardiac autonomic modulation in patients with MS.

Resting HR was not different between groups, in contrast to previous results reported in diabetic patients (Baldi et al., 2016) and corroborating the results found in MS (Alihanoglu et al., 2015). The impaired cardiac parasympathetic modulation in the absence of sympathetic hyperactivity also contrasts with previous studies in diabetes (Facchini et al., 1996; Schroeder et al., 2005). Therefore, one novel finding of the present study is that resting HR and cardiac sympathetic modulation do not seem to be compromised in the initial stages of MS. Another interesting finding was that MSG presented an altered cardiac autonomic modulation, as compared to CG.

In addition to the impaired cardiac parasympathetic modulation at rest, MSG had a slowed HR response during the on-transient, which was associated with a slowed parasympathetic withdraw. Currently, we are not aware of any other study that has investigated kinetics during exercise in MS. The HR response during on-transient in the dynamic exercise results in a cardiac adjustment to supply the energy demands of the muscles involved (Rowell and O'Leary, 1990). Therefore, a slowed on-transient might impair exercise performance due to an inadequate blood supply to the working muscles. In line with this, our results showed that MSG reported higher RPE for the legs, but not for dyspnea when compared to CG. In addition, all participants interrupted the test before reaching a plateau in oxygen consumption, which is assumed to confirm that maximal oxygen uptake was not attained during incremental exercise to fatigue, thus suggesting that peripheral fatigue was the major determinant of test performance.

Regarding the off-transient responses, the present findings showed that MSG presented slower HRR compared to CG, which seems to be due to a slowed cardiac parasympathetic reactivation at the end of exercise. HRR after exercise was related to the high frequency power, which confirms that the off-transient is mainly a function of a reactivation of the parasympathetic nervous system (Arai et al., 1989; Imai et al., 1994). These findings are in accordance with those reported by Simões et al. (2013), that found a slower HR and VO2 kinetics in healthy elderly subjects compared to the young group for off-transients, and suggested that impairment of cardiac autonomic modulation was the main underlying mechanism. Earlier studies reported that the low frequency power is a major predictor of later cardiovascular events (Tsuji et al., 1994). In addition, an impaired HRR after exercise has been shown to be a strong predictor of overall mortality, independently of workload, changes in HR during exercise and the presence of myocardial perfusion disturbances (Lind and Andren, 2002). Moreover, a decreased HRR is one possible mechanism by which MS is associated with increased cardiovascular disease morbidity and mortality (Sattar et al., 2003). Therefore, the present confirmation of a slowed off-transient is an important clinical finding in MS (Lind and Andren, 2002; Spies et al., 2005; Alihanoglu et al., 2015).

A further important finding is the absence of significant correlations between the altered HR responses with WC and BMI accompanied by a significant correlation of all the altered parameters with fasting blood glucose, which suggests that measurement and control of blood glucose might play a central role in MS management. The 3.3 ml/kg.min^−1^ (~1-MET) difference in mean exercise capacity between MSG and CG might translate into substantially increased mortality for people with MS, since previous authors reported that every MET decrease in exercise capacity decreases the likelihood of survival by 12% (Myers et al., 2002; Kodama et al., 2009).

In summary, we found that MS is associated with poor exercise capacity and poor heart rate kinetics. The altered HR kinetics seems to be related to alterations in parasympathetic modulation, and glucose metabolism seems to be their major determinant. Many hypotheses have been raised to explain the attenuated HR response to exercise in the face of insulin resistance, as central sympathetic signaling to the heart; impaired beta-adrenergic responsiveness; and the intracellular events that stimulate/inhibit systolic work (Baldi et al., 2016). Although the present study cannot exclude the other factors, it suggests that parasympathetic modulation has an important role in MS. Autonomic modulation dysfunction is identified in the initial stages of diabetes or even precedes its diagnosis, while the reductions in beta-adrenergic responsiveness and alterations in contraction-relaxation coupling might be a consequence of chronic exposure to high levels of glucose (Baldi et al., 2016). Therefore, these mechanisms might be more evident in people with diagnosed diabetes than in MS.

Parasympathetic nervous activity has its greatest influence on heart rate, with only moderate effects on heart contractility (Landzberg et al., 1994), which can explain the slowed off-transient (Fang et al., 2005), but not the slowed on-transient. The attenuated parasympathetic response would result in higher HR during stress, but the response found in the present study was a reduced and slowed increase in HR. Further studies should be conducted to investigate the mechanisms involved.

Despite interesting results, some limitations must be pointed out. Exercise capacity and HRR were measured during a treadmill test that relied on a participant's effort to reach maximal effort, which we attempted to overcome by employing experienced personnel to motivate participants. In addition, the level of habitual physical activity was not measured in the present study, so we could not quantify the contribution of this variable on the studied outcomes. Thus, considering that sedentary lifestyle is usually a factor contributing to MS, future studies should address this issue in order to quantify its impact on HR kinetics and cardiac autonomic control. Other point that should be mentioned is the higher number of women on climacteric period in the MS group compared to the CG. However, in order to account for a possible influence in our results, an ANCOVA was performed controlling the between groups comparisons for age and being/not being on climacteric period. The results remained unchanged, thus excluding these variables as confounding factors. Moreover, the present results are based on a cross-sectional analysis, so we cannot determine the causal direction between the metabolic syndrome, autonomic modulation and exercise capacity.

Despite these limitations, this study may have important clinical implications and is a starting point for future studies Considering that the primary targets of therapy for MS are lifestyle changes, such as adjusting nutritional habits and increasing physical activity (Golbidi et al., 2012; Roberts et al., 2013; De Sousa and Norman, 2016), the present results have many important practical applications for health professionals involved in the care and management of MS. The slow on-transient suggests that exercise should be preceded by an adequate warm-up and there should be a controlled progression of exercise intensity in order to allow for metabolic and cardiac adjustments. Although high intensity has been suggested for this population (Aguilera Eguia et al., 2015; Baldi et al., 2016), the performance of high intensity short duration sprint interval training might be limited by peripheral factors and, consequently, might not provide the necessary cardiac adaptations. Therefore, protocols performed at lower intensities for longer durations, such as high intensity aerobic interval training, might be a better choice, at least in the initial phases of exercise prescription. Finally, the slowed off-transient implies that exercise should be proceeded by a supervised cool-down period in order to reestablish cardiac function in MS patients.

## Conclusions

In conclusion, our findings showed that women with MS presented low exercise capacity and poor on- and off-HR kinetics compared with women without MS. Furthermore, cardiac parasympathetic modulation impairment was suggested to be the main variable underlying such alterations and fasting glucose levels were the only independent variable explaining the impaired cardiac autonomic modulation in patients with MS.

## Author contributions

LS and AR collected data, wrote, reviewed and edited the manuscript. AZ contributed with data analysis, wrote, reviewed and edited the manuscript. PG wrote, reviewed and edited the manuscript. FA collected data and contributed with the discussion. AL collected data and contributed with data analysis and discussion. VS collected and analyzed data. MS collected data and reviewed the manuscript. MV contributed with data analysis. KS contributed with data analysis and reviewed the manuscript. GP contributed with data analysis and reviewed the manuscript.

### Conflict of interest statement

The authors declare that the research was conducted in the absence of any commercial or financial relationships that could be construed as a potential conflict of interest.
